# A Qualitative Exploration of the Experiences and Perceptions of Interpersonal Relationships Prior to Attempting Suicide in Young Adults

**DOI:** 10.3390/ijerph19137880

**Published:** 2022-06-27

**Authors:** Heather McClelland, Jonathan J. Evans, Rory C. O’Connor

**Affiliations:** Institute of Health and Wellbeing, University of Glasgow, Admin Building, Gartnavel Royal Hospital, 1055 Great Western Road, Glasgow G12 0HX, UK; jonathan.evans@glasgow.ac.uk (J.J.E.); rory.oconnor@glasgow.ac.uk (R.C.O.)

**Keywords:** suicide, loneliness, qualitative, interpersonal, social

## Abstract

Suicide is a leading public health concern. Research studies have identified significant associations between loneliness and suicidal ideation/behaviour both cross-sectionally and prospectively. Despite this, research specifically focusing on identifying the nature of loneliness experienced prior to suicide, and the role it has in association with other preceding factors, has not been fully explored. The current study recruited ten participants with a history of suicide attempts (five female, four male and one non-binary; mean age: 22.5, range: 20–25 years) to take part in one-to-one, semi-structured interviews via remote video conferencing to explore experiences of social support and loneliness prior to suicide attempt. Using Interpretative Phenomenological Analysis, several aspects of loneliness emerged as important themes that were present prior to participants’ suicide attempts. Additional themes identified were patterns of social support, personality traits, emotional secrecy and social transition. Evidence suggests that a positive relationship with parents, knowing someone with similar experiences or having membership in more than one friendship group may reduce feelings of loneliness and/or intentions to die. This research makes an important contribution to understanding the role of loneliness in relation to suicide attempts by highlighting the importance of social supports being emotionally available to those experiencing distress.

## 1. Introduction

Suicide accounts for approximately 700,000 deaths worldwide every year [1]. However, the aetiology of suicide is both multifaceted and complex. In recent years, there has been increased focus on the psychological determinants of suicide risk [2]. To this end, one such factor that is receiving growing recognition is loneliness [3,4,5,6]. However, not everyone who is lonely attempts suicide, and not all who become suicidal are lonely. Therefore, it is important to better understand the role of loneliness in suicide behaviour.

Loneliness is acknowledged as an important factor within Joiner’s Interpersonal Theory of Suicide (IPT; [7]). Within the IPT, loneliness is considered a component of thwarted belongingness, and when both thwarted belongingness and perceived burdensomeness occur at high levels, Van Orden et al. (2010) argue that suicidal ideation is likely to emerge. Similarly, the Integrated Motivational Volitional model (IMV) [8,9] includes thwarted belongingness, in addition to other socially oriented contextual (e.g., social support) and predispositional (e.g., perfectionism) factors in a multifaceted, biopsychosocial model of suicidal behaviour. In doing so, the IMV model considers both the individual’s past and present experiences and perceptions when predicting suicidal behaviour. Unlike the IPT, however, the IMV model does not currently explicitly include loneliness within its model, although it does include belongingness and social support.

Loneliness is a broad construct [10], but a widely accepted definition is that it reflects a discord between the quality or quantity of relationships an individual has and those that they want [11]. Furthermore, Weiss (1973) proposed that social loneliness (e.g., friendships) is distinct from emotional loneliness (e.g., romantic partners, family) [12]. The health risks associated with loneliness are addressed by Cacioppo and Hawkleys (2009) evolutionary approach [13]. Cacioppo and Hawkley posit that short-term loneliness can be helpful in prompting lonely individuals to seek-out new bonds or reinforce existing ones, while long-term loneliness can lead to physical and psychological vulnerability, including possible depression and death [14]. Evidence to support this was found in a systematic review and meta-analysis by McClelland et al., (2020), where loneliness was associated with suicidal ideation and behaviour between 10 weeks and 5 years after reports of loneliness [3]. Furthermore, this association was strongest in young adults and female participants. Although this review helped to identify at-risk populations who may be particularly susceptible to developing suicidal ideation and behaviour following loneliness, it did not identify which aspects of loneliness contribute to suicidal behaviour. The present study used qualitative research methodology, therefore, to examine which aspects of loneliness are most influential in driving suicidal ideation/behaviour.

Qualitative research can provide valuable insights into human behaviour. For example, Interpretative Phenomenological Analysis (IPA) uses an idiographic approach to conduct in-depth analyses of a specific event based on first-hand accounts [15]. Such approaches can help to understand the significance individuals attach to events [16], and therefore, common themes can be identified between narrative accounts.

No published, peer-reviewed studies have used IPA to explore loneliness prior to suicidal behaviour. However, research by Lee, Vasileiou and Barnett (2019) identified aspects of loneliness in new mothers, including unfavourable self-comparisons, social isolation and unempathetic relationships [17]. This demonstrates that using IPA is an effective approach to exploring loneliness in relation to significant life events. IPA may provide valuable insights into understanding how loneliness may be a factor in those who later choose to take their own lives. Doing so may help improve clinical and community guidance for suicide prevention.

### Current Research

The current study used IPA to explore narrative first-hand accounts of the process that led to participants’ decision to engage in suicidal behaviour and the social supports participants believe they had available to them prior to their attempt. The study also explored participants’ experiences of loneliness and whether the latter was a factor in their decision to end their life. To that end, the study addressed the following two research questions:

1. What role, if any, does the availability and quality of social supports have on wellbeing prior to a suicide attempt?
2. What role, if any, does loneliness have on one’s decision to attempt suicide?

It is hoped this research will improve understanding of the role of loneliness in the emergence of suicidal ideation and behaviour, thereby informing the development of suicide prevention strategies.

## 2. Materials and Methods

### 2.1. Procedure and Interview

The study was conducted in accordance with the Declaration of Helsinki, and the protocol was approved by the Ethics Committee of the University of Glasgow’s College of Medical, Veterinary and Life Sciences (reference no.: 200190125). Screening and recruitment took place between September and December 2020. Study advertisements were circulated via the University of Glasgow participants pool on social media sites, and snowballing advertising strategies were applied. Prospective participants contacted the lead researcher via email or telephone call to register their interest in taking part. All prospective participants were required to complete a screening telephone interview to ensure participants’ eligibility.

Eligible participants for this study were: ≥18 years old, had made a suicide attempt within the last five years, had sufficient proficiency in English to complete the interview and had access to a computer microphone. Participants were excluded if they indicated they had cognitive impairment or had experienced symptoms of psychosis within the last two weeks.

Eligible participants were invited to participate in the study interview, with the date and time of the interview arranged during the screening call. Study interviews were conducted via video conferencing (Zoom or Microsoft Teams) chosen by participants. Interviews were conducted by the lead researcher, and participant experiences followed the following four-part structure:

1. Review of the participant information sheet, privacy notice and consent form. Consent was audio recorded.
2. Socio-demographic information and health history data (see Section 2.2) were collected verbally by the lead researcher and recorded in an Excel database.
3. Audio-recorded semi-structured interview.
4. Online completion of psychometric assessments (using University of Glasgow Online Survey Systems; reported elsewhere for the purposes elsewhere as part of a doctoral thesis).

Webcams were not required for this study but could be used at the participants’ discretion. Seven out of ten participants elected to use their webcams. The researcher’s webcam remained on throughout the interaction, but the researcher invited participants to advise if they preferred the camera to be turned off (*n* = 0). Participant interviews lasted between 17 and 52 min, and video conferencing continued between the researcher and participant to resolve any queries or issues during the online survey. Participants were compensated with GBP 25 Amazon vouchers for their time. All data were stored on a password-protected computer at the University of Glasgow. Participant interviews were transcribed using a third-party transcription service. Following standardised transcription-to-written formats, audio files of the semi-structured interviews were permanently deleted.

### 2.2. Data Collection

Demographics. Participants’ age, education history, relationship status, sexual orientation, living status, mental health history and suicidal behaviour history were collected.

Semi-structured interview. The interview schedule can be found in Appendix A. Follow-up questions were asked to explore responses in greater detail, where appropriate. The final interview question (‘Many people feel lonely within their lives; are there any times you have felt particularly lonely?’) was only posed if the participant had not mentioned loneliness in their previous interview responses.

### 2.3. Qualitative Analysis

Interpretative Phenomenological Analysis (IPA) allows detailed analysis of lived accounts of a specific event and affords insight into a person’s interpretation of a specific event, with exploration of the significance of that event. Consistent with Creswell [18] (p. 161), ten participants is considered a sufficient number for studies of this nature. Following Smith and Shinebourne (2021), a line-by-line analysis of the interview transcripts was conducted to identify themes [19]. These themes were then grouped to identify superordinate themes, which were reviewed by all research authors for accuracy and applicability.

### 2.4. Participant Characteristics

Participants submitted notes of interest in the study. The first 14 potential participants completed the screening call, of which 10 completed the study interview. Ineligible participants (*n* = 4) identified during screening were excluded due to experiences of psychosis in the last two weeks (*n* = 2), no history of suicide attempts (*n* = 1) and insufficient proficiency in English (*n* = 1).

All participants were allocated pseudonyms to protect their identities. Participant socio-demographic characteristics are summarised in Table 1. In summary, half of participants were female (*n* = 5) and most were White (*n* = 8), bisexual (*n* = 6), single (*n* = 9) and lived with flatmates (n = 8). All participants were young adults (age range at the time of interview: 20–25 years). On average, participants’ last suicide attempt was 2.46 (sd. 1.47) years prior to their participation in the study.

## 3. Results

Five superordinate themes and ten sub-themes were identified across the ten interviews (see Table 2). An explanation of the attributed theme name and excerpts from transcripts reflecting the theme are provided under each heading below. Additional excerpts from other participants can be found in Supplementary Materials Section S2.

### 3.1. Patterns of Social Support for Self-Worth

All participants talked about their experiences of seeking social support prior to their suicide attempt. Three sub-themes were also identified: participants describing their relationship with their parent(s); a preference for non-familial support; and a strong reliance on one person.

#### 3.1.1. Relationship with Parent(s)

Seven participants indicated a difficult relationship with a parent prior to their suicide attempt. Below, Rachel outlines how her relationship with her parent influenced her belief about all other relationships she had, which influenced her self-worth prior to her attempt.

‘… It obviously did have a big impact, and it’s kind of, it affects all your other relationships because the one person who’s meant to accept you for everything and careabout you or whatever, just wasn’t there. It’s kind of like, if she doesn’t care about me, why would anybody else?’

(Rachel, 20 years)

Rachel describes how the lack of their mother’s care and affection led them to believe that no one else would care about them, thereby alluding to feelings of despair, defeat and diminished sense of belonging. All participants who indicated having a negative relationship with their parent(s) indicated that these experiences adversely affected their mental health (see Supplementary Materials Section S1.1 for further examples).

#### 3.1.2. Preference for Non-Familial Support

Six participants stated they were more likely to share, or allude to, their mental health with a friend rather than a family member, as summarised by Aoife below:

‘…I feel close to my friends. I’m quite close with my mum but I don’t talk to her about my mental health.’

(Aoife, 24 years)

Aoife explains that she censors her disclosures to her mother, while she did not mention any caveats about what she would potentially discuss with friends. This suggests a possible preference for support-seeking from friends instead of family when in psychological distress, which was also evident in other interviews (see Supplementary Materials Section S1.2).

#### 3.1.3. Strong Reliance on One Person

Six participants indicated they were particularly reliant on one individual (e.g., parent, romantic partner). Take Trevor’s experience:

‘…. My first girlfriend, I put everything into it, and when I put everything into it, I distanced myself from other people. So, when the connection wasn’t perfect between both partners, that inspires a real sense of loneliness, that this person should get me.’

(Trevor, 22 years)

Trevor reflects how, by focusing on his romantic relationship, his bonds with other social networks were weakened through neglect. When his romantic relationship became strained, Trevor described how this led to feelings of loneliness. This could be because there was nobody else he could rely on for support or due to romantic loneliness (see Supplementary Materials Section S1.3 for more examples).

### 3.2. Aspects of Loneliness

All ten participants indicated that at least one aspect of loneliness contributed to their decision to die. Therefore, all emergent aspects of loneliness are discussed below with sub-theme names based on those identified by Rosedale (2007), which were considered to fit well with the aspects of loneliness described by participants.

#### 3.2.1. Social Isolation

Isolation, both voluntary and involuntary, was described in six interviews; take Chloe, for example:

‘… I came to uni and sort of like, you know, it was like a restart button like I again, felt very isolated, I guess. Yes, I guess talking about loneliness, in the first year [of university], the first couple of months I didn’t know anybody, I didn’t get on with the flatmates and being in a different country and just a new city and everything.’

(Chloe, 23 years)

Chloe describes involuntary social isolation as she was enthusiastic about building friendships, but in her new environment, this had proven challenging, and she had been unsuccessful in building friendships with her flatmates. As stated by Cacioppo and Hawkley (2009), short-term loneliness can incentivise people to strengthen bonds, but if unresolved, it can lead to mental illness in the long term (see Supplementary Materials Section S2.1 for more examples).

#### 3.2.2. Lack of Emotional Connectedness

Emotional connectedness describes the individual’s belief they have an emotional, reciprocal bond with those within their social network and surfaced in seven interviews. Mohammad’s account was most salient of all:

‘… I wasn’t feeling that greatly connected at that point in time. So it all added more fuel to me, you know? ‘Cause even as I was planning to... I was not, you know, so much attached to them, no matter how attached and how warm they were to me.’

(Mohammad, 25 years)

Mohammad indicates that he was aware of his family’s affection for him; however, he felt unable to reciprocate this prior to his suicide attempt, which ‘added more fuel’ for his suicide attempt. This suggests this lack of connectedness was a particular risk factor for suicide for Mohammad, though others also reported a lack of emotional connection around the time of the suicide attempt (see Supplementary Materials Section S2.2).

#### 3.2.3. Lack of Feeling Understood

Five participants reported not feeling understood by those around them, with this negatively affecting their wellbeing. Taj illustrates this below:

‘… I was always surrounded by people but I thought they never really, sort of, understood me and that would’ve contributed to, sort of, the feeling of helplessness or, you know, I don’t fit in here kind of thing.’

(Taj 21 years)

Taj identified his experience of not feeling understood by those around him as a contributing factor to his feeling of helplessness. During his interview, Taj shared that he disclosed some feelings to his friends; however, his attempts to gain understanding from others were thwarted. These consequential feelings of helplessness that Taj described are not dissimilar to entrapment, a factor strongly associated with suicide (see Supplementary Materials Section S2.3 for other examples).

### 3.3. Emotional Secrecy

Emotional secrecy is the deliberate concealment of emotion from others [20] and includes sub-themes of anticipated stigma and autonomy. Emotional secrecy arose in seven interviews. Aidan’s example is below:

“…I think I would say that I always played it down and always…If, so I would either hide it completely and just keep it to myself or if I did tell people then I would play it down a lot.”

(Aidan, 20 years)

The effort Aidan made to conceal his distress is apparent in the quote above, where Aidan describes that he would either conceal it entirely or actively hide the severity of his distress. As those around him did not know the extent of his suffering, Aidan would have gained little or no social support for his emotional turmoil, which increased his propensity for suicide (see Supplementary Materials Section S1.3).

#### 3.3.1. Anticipated Stigma

Anticipated stigma was a prevalent sub-theme in six interviews. Below, Margaret describes her anticipation of negative stigma should she disclose her mental health difficulties to her friends.

‘… I knew I had lots of people that I spent time with, and that there were people that I could rely on to text and be, like, do you want to go to the pub, let’s grab a drink, let’s go and do this. But I didn’t feel like they were people that would necessarily stay my friend if they knew that I had mental health problems or if they knew I was suicidal.’

(Margaret, 25 years)

Despite having an established and dependable network to socialise with, Margaret describes how she felt these friendships would not withstand her disclosure of poor mental health. Margaret does not justify why she had these beliefs of negative stigma; instead, this appears to be an assumption she has made about her friends (see Supplementary Materials Section S3.1 for other examples).

#### 3.3.2. Autonomy

Concern for autonomy has consistently been associated with avoidance or disengagement with health services and increased the propensity for suicide [21]. Five participants indicated a need for autonomy, as illustrated by Elsie:

‘… I might have told people that weren’t living with me [that I was feeling suicidal] ‘cause, as I say, it’s the people that are living with me that could have intervened more. So, if they could have intervened, I wouldn’t have told them [what I was doing/planning].’

(Elsie, 21 years)

Elsie illustrates that disclosing her distress to others was determined by logistics and may have considered telling people who would not have been able to hinder her intentions. This suggests that although Elsie may have wanted to make others aware of her suffering, at that point, her intention to die was strong, and she was not looking to be saved (see Supplementary Materials Section S3.2 for other examples).

### 3.4. Personality Traits

Personality characteristics influence how individuals navigate and respond to social interactions [22]. Descriptions consistent with two personality factors emerged from these interviews: socially prescribed perfectionism and an anxious disposition.

#### 3.4.1. Socially Prescribed Perfectionism

Socially prescribed perfectionism (SPP) is the need to meet the real or perceived unreasonably high expectations an individual believes others have for them [23]. SPP was evidenced in half of the interviews (*n* = 5), including in Aidan’s interview.

‘… It was partly like even though I was doing well in school, I didn’t see that, I didn’t perceive that because like my mum was telling me that I wasn’t … I was a big perfectionist because of that so I felt like everything I did, if it wasn’t absolutely perfect then it wasn’t, then it was awful and I was a failure.’

(Aidan, 20 years)

Aidan described his mother as being critical and having high expectations of his academic achievements. Despite his own beliefs, it appears from this quote that Aidan may have internalised his mother’s views, and therefore, when Aidan ‘wasn’t absolutely perfect’, he automatically criticised himself (‘I was a failure’) and perpetuated his mothers’ standards from within. SPP adds an extra element of stress beyond the typical daily stressors and is therefore consistently associated with suicidal ideation and behaviour (see Supplementary Materials Section S4.1 for other examples).

#### 3.4.2. Anxious Disposition

Anxiety can occur when someone is overly concerned with their welfare, to the point that it impedes day-to-day living in some way. Seven participants indicated an anxious disposition; commonly, this was linked with academia, as illustrated by Chloe below.

‘… I got the results from university that I was progressing and it was a relief but it was also like it caused me to be even more anxious because I was thinking, if I struggled for this year, how am I going to manage the next year?’

(Chloe, 23 years)

Chloe describes an automatic dismissal of her achievements and instead fixates on the potential for failure the following year. This illustrates Chloe’s ingrained anxiety, which decreases one’s ability to manage additional stress and consequently increases the opportunity for defeat and potential later suicidal behaviour (see Supplementary Materials Section S4.2 for other examples).

### 3.5. Social Transition

Instances of social transitions were identified in five interviews, including moving away from home to begin university (*n* = 4) and, in Rachels’ case below, changing schools within the same geographic area:

‘…2016 was a weird year because the first eight months were probably like the best time for ages. I was like, you know, confident, obviously I was doing GCSEs then, things were going pretty well. Then I moved to sixth form [college] and I think things just kind of reverted back to anxiety, depression, all this stuff.’

(Rachel, 20 years)

Rachel recalls a marked deterioration in her mental health as she began sixth form, with her new surroundings preceding negative affective states associated with increased risk of suicide. Social transitions can lead to stress as one adjusts to their new surroundings, which often co-occurs with a loss or reduction in social supports. These additional stressors can make daily hassles more difficult to manage and increase the potential for suicidal behaviour (see Supplementary Materials Section S5 for other examples).

## 4. Discussion

This is the first study to use IPA to investigate experiences of social support and loneliness prior to a suicide attempt. Although individual differences were identified across the interviews, three aspects of loneliness were identified from the interviews: social isolation, lack of emotional connectedness and not feeling understood. Four additional overarching themes emerged: patterns of social support, emotional secrecy, personality characteristics and social transition. Most participants reported a detachment from those around them (e.g., lack of emotional connectedness, social isolation) prior to their suicide attempts and most indicated they would, or did, prefer to speak to their friends about their mental health instead of their family members.

The findings suggest that those who had a strong reliance on one person for social support may have felt loneliness more acutely than those who maintained broader social networks. All participants who relied on one person, often a romantic partner, reported that their wider support networks did not compensate for the loss of this specific bond. This is consistent with Weiss’s (1973) theory of loneliness [12]. Weiss argued that emotional loneliness, which arises through the absence of a close bond, cannot be overcome by the presence of other social supports. The findings also fit with both the IMV model of suicidal behaviour [8] and the IPT [7]. The IMV model posits that the absence of social support can increase the likelihood of entrapment, giving rise to suicidal ideation. Research has shown that loneliness may operate in a similar fashion where, within the context of the IMV, the presence of loneliness may increase the likelihood that people who feel trapped become suicidal [24]. Equally, the IPT suggests that loneliness contributes to feelings of thwarted belongingness, which, in the presence of perceived burdensomeness, is also posited to lead to suicidal ideation.

Aspects of loneliness, as identified within the current study, are consistent with findings by Sjöberg et al. (2018) [25]. Specifically, when exploring feelings of loneliness in older adults with a history of suicide attempt, Sjöberg et al., (2018) suggested that loneliness commonly stemmed from feeling disconnected or alienated from significant people in their lives (i.e., romantic loneliness) and a lack of feeling understood. Although these themes also emerged in the current study, the origins of loneliness were not explored here.

Another theme to emerge from the current study was the participants’ relationship with their parent(s). The interviews indicated that the quality of one’s current relationship with their parent(s) affected their mental health, as participants would generalise their parental relationship to other relationships. This is consistent with research from attachment theory in early life [25,26] and bonds in adults with their parents finding significant associations with mental health [27,28,29], as indicated by the first-hand accounts of the young adult group of the current study.

The potential effect of parental attitudes on wellbeing was evident in the context of the SPP theme. This fits with Hewitt and Flett (1990), who found that early life experiences (e.g., parental expectations and concern over mistakes) can lead to the manifestation of perfectionistic behaviours in children where they are driven to meet the real or perceived expectations that they believe others hold for them [23]. Due to their belief that their relationships are superficial, individuals may perceive their key relationships as being insecure [30] and thereby potentially instil a sense of loneliness [31,32]. Alternatively, those who are able to maintain a persona of perfection may develop superficial relationships, which leads to feelings of loneliness to arise as they do not assume social acceptance [33,34].

Equally, emotional secrecy was described frequently during the interviews, which perhaps serves to support their need to maintain a persona of perfection or else risk being rejected by their friends.

The findings also reinforce Perlman and Paplau’s (1982) argument that the quality, not just the quantity, of one’s connections must be considered when assessing loneliness. Social support networks are consistently posited to protect against suicide risk; however, this is often based on quantity or existence of connections (e.g., marital status, number of children, number of flatmates), therefore overlooking whether these contacts are people the individual would choose to reach out to [11,35]. This is illustrated by the sub-theme of ‘relationship with parent(s)’ in the current study. Individual differences, including personality traits, quality of parental relationships and patterns of support, must be considered when attempting to identify whether one’s social support network might help keep an individual safe from suicide.

This research highlights the distinction between perceived loneliness (i.e., loneliness as a psychological state) and actual loneliness (as a consequence of the interpersonal or social environment), as illustrated by the sub-themes of social isolation and lack of emotional connectedness [36]. Despite similarities between the themes of emotional loneliness and social isolation, it is possible to experience one without the other, or conversely, one may induce the other (e.g., feelings of loneliness leading to social withdrawal and self-imposed isolation). However, how these may differ in their impact on suicide risk warrants further exploration. Indeed, the current study also identified the theme of anticipated stigma; however, how this compares to public stigma (i.e., stigmatising views explicitly declared by those around the individual) in the manifestation of suicidal behaviour also warrants investigation.

### 4.1. Implications for Theory

The findings of the current study support Joiner and van Ordens IPT by explicitly highlighting the adverse role loneliness can potentially have on the development of thwarted belongingness and possible suicidal ideation. Equally, the IMV model also includes thwarted belongingness as an antecedent of suicidal ideation. However, the findings of the current study indicate the potentially distinctive role romantic loneliness might have above other forms of social support or marital status. Furthermore, factors associated with emotional secrecy are not highlighted in either model. Despite social norms being included in the IMV model, neither framework explicitly addresses the role of stigma or autonomy. Such factors, however, are well-established correlates of mental wellbeing.

### 4.2. Limitations

All interviews were conducted online due to the social distancing requirements imposed during the COVID-19 pandemic. The online administration directly affected the flow of conversation in two of the participant interviews, which were disrupted due to internet connection difficulties. The sample was comprised exclusively of young adults, and as such, the findings cannot be generalised to middle or older age groups. Equally, over half of the participant group was bisexual, which does not reflect the prevalence in the general population. Another consideration is that although romantic relationships ended for several participants around the time prior to their suicide attempts, the romantic status and quality of those relationships of the remaining participants at the time of their suicide attempt were not explored. Finally, there was considerable overlap in themes that we were unable to explore within the parameters of this paper. Given this overlap, further in-depth investigation is required to help to disentangle any cause-and-effect relationships between the themes identified here and suicide attempts.

### 4.3. Future Work

This is the first peer-reviewed publication to use IPA to investigate the association between interpersonal factors (specifically loneliness) and a suicide attempt drawing from accounts of individuals with lived experience. The findings from this study, however, provide the basis for further investigation into the relationship between romantic loneliness, parental relationship (both past and present) and suicidal behaviour. Future work would also benefit from exploring differences between anticipated and public stigma.

## 5. Conclusions

In conclusion, this is the first study to use IPA to explore experiences of social support and loneliness in the context of understanding the factors that may lead to a suicide attempt. Several aspects of loneliness, in addition to other interpersonal factors such as emotional secrecy and patterns of support, emerged as significant in young people. The evidence here suggests that acceptance, especially from significant others (e.g., parents, romantic partners), may play a pivotal role in mental wellbeing. These findings offer preliminary qualitative evidence that is consistent with the IMV model and IPT models, where the quality of one’s social supports and feelings of belongingness may be associated with the emergence of suicidal behaviour. Future research may benefit from exploring the role of parental attachment style and romantic loneliness in the pathways to suicidal behaviour longitudinally.

## Figures and Tables

**Table 1:** Participant characteristics.

	Aoife	Trevor	Chloe	Elsie	Rachel	Margaret	Alice	Aidan	Taj	Mohammad
Gender	Female	Male	Female	Female	Non-binary	Female	Female	Male	Male	Male
Age	24	22	23	21	20	25	24	20	21	25
Ethnicity	White	White	White	White	White	White	White	White	Asian	Asian
Sexuality	Bisexual	Heterosexual	Bisexual	Bisexual	Pansexual	Bisexual	Bisexual	Bisexual	Heterosexual	Heterosexual
Relationship status	Single	Single	Single	Single	Single	In a relationship	Single	Single	Single	Single
Living arrangement	Lives alone	Flatmate(s)	Flatmate(s)	Flatmate(s)	Flatmate(s)	Live with partner	Flatmate(s)	Flatmate(s)	Flatmate(s)	Flatmate(s)
Most likely to confide in	Friend(s)	No one	Cousin	Friend(s)	Mother	Friend(s)	Friend(s)	Friend(s)	Friend(s)	No one
No. suicide attempts	15	5	2	‘Too many to count’	1	3	4	3	2	2
Nature of latest attempt	Impulsive	Planned	Impulsive	Impulsive	Planned	Impulsive	Impulsive	Unclear	Planned	Planned

**Table 2:** Themes and subthemes identified.

Theme	Sub-Theme		
Patterns of social support	Relationship with parent(s)	Preference for non-familial support	Strong reliance on one person
Aspects of loneliness	Social isolation	Lack of emotional connectedness	Lack of feeling understood
Emotional secrecy	Anticipated stigma	Autonomy	
Personality traits	Socially prescribed perfectionism	Anxious disposition	
Social transition			

## Data Availability

The data presented in this study are available on request from the corresponding author. The data are not publicly available due to the specifications outlined by the ethical committee which approved this study.

## Supplementary Materials

The following supporting information can be downloaded at: https://www.mdpi.com/article/10.3390/ijerph19137880/s1.

## Appendix A. Interview Questions

**Open Question**	**Probing Question**
With your last suicide attempt in mind, can you describe what going on in your life?	- What patterns or triggers do you associate with any thoughts of suicidal ideation or behaviour you experienced at the time? - Why do you think you started to experience suicidal ideation/engage in suicidal behaviour? - Did you notice any patterns that you felt were linked to your suicide behaviours? These could be thoughts or feelings you had, behaviours or certain situations. - What did you want to achieve by dying?
How do you remember feeling about your friends and family at the time?	- Who would you turn to when you were feeling down or wanted to talk about your thoughts of suicide? - Did your thoughts of friends and family influence your want to die in any way?
To be asked only if the participant has not mentioned loneliness within their previous answers:	
Many people feel lonely within their lives; are there any times you have left particularly lonely?	- Does this relate to your experiences of suicide in any way?
