# Supplementary material for: A Qualitative Exploration of the Experiences and Perceptions of Interpersonal Relationships Prior to Attempting Suicide in Young Adults
Source: Int J Environ Res Public Health. 2022 Jun 27;19(13):7880. doi: 10.3390/ijerph19137880 (PMC9266001; doi:10.3390/ijerph19137880)
Supplement: Supplementary file 1 [file ijerph-19-07880-s001.zip › ijerph-1741069-supplementary.pdf]

Supplementary material. Further supporting quotes for each theme and sub-theme.

*1. Patterns of social support*

1.1. *Relationship with parent(s)*

Negative relationship with parent

*"…If you expressed any kind of emotional suffering, she would just like either get angry at you or make a joke out of it. Um and, yea, just like a really not supportive person at that time*." (Aidan, 20 years)

Aidan describes his thwarted attempts to seek emotional support from his mother. Instead of support and reassurance, Aidan illustrates how his mother discredits or invalidates Aidan's feelings. In the above quote, Aidan stresses that he was particularly aware of his mother's behaviour in the lead-up to his suicide attempt.

Positive relationship with parent

*"…I would be thinking of them as sort of like an anchor rather than something that makes the situation worse."* (Chloe, 23 years)

In contrast to Aidan, Chloe describes how she viewed her relationship with her parents positively and as a source of support. She compares her parents to an anchor, possibly grounding her and reminding her of her value. As such, this suggests that positive relationships between participant and parent buffer someone considering suicide.

1.2. *Preference for non-familial support*

*'… I'm not particularly close to my family and I'm an only child as well, I don't have siblings, so I've always relied...for as long as I can remember, always relied on the friends that I choose to, sort of, be my family and be my, sort of, support structure.'* (Taj, 21 years)

Above, Taj (21 years) explains his preference for support from his friends. He justifies this as being resultant of having no siblings to confide in and having a lack of emotional proximity with his family. Taj later reinforces this in the interview with the following comment:

'*As far as I can remember I've always relied on my friends to, sort of, be my support structure as opposed to my parents*'

Therefore, it may be that Taj relies on the support of his friends as a last resort, as opposed to an initial preference. During the interview, Taj explains that thoughts of suicide emerged following the loss of his support structure.

1.3. <u>Strong reliance on one person</u>

*"…Like, when we broke up, losing that support I think is probably what kind of tipped me over the edge a little bit, because I didn't really know who to tell".* (Margaret, 25 years)

The shortcomings of relying heavily on one person are made salient by Margaret (25 years). Here, Margaret does not indicate any sadness or longing for her partner specifically, but instead, the role that they played in supporting her wellbeing and acting as a confidante. Through the absence of her former partner, Margaret became aware of a lack of others to seek support from, which she describes as a contributing factor to tipping her 'over the edge'.

*<u>2 Aspects of Loneliness</u>*

2.1 *Social isolation*

*"…So my support network wasn't that strong. I did have quite a few friends in Ireland, but I'd moved away from Ireland, like I was the only one in my school that did move to a different country. And also, one of the people I was closest with, he passed away on the final day of my leaving cert examinations. So I was…like it shook the nature of friendship for me… I had built a foundation with him."* (Aidan, 20 years)

Here, Aidan describes social separation in two forms; moving away from home and the death of a friend, both of which happened in quick succession. Aidan states that due to moving from home, his support network was weak, thereby suggesting that he believed the strength of his support network was dependent on physical proximity. Furthermore, he describes the pain of the death of his friend, 'I had built a foundation with him', which suggest a fundamental means of social support for Aidan was gone.

*2.2 Emotional connectedness*

*"…I have a good group of friends, like, quite a few different groups of friends. I have a good relationship with my family. But in those instances, and those periods of my life, I felt that I was very detached from them*." (Alice, 24 years)

Alice describes a shift in their social connectedness towards all friendship groups and their family. They suggest here that they felt connected before and after their desire to die, but during this time, this connectedness was lost, and they felt 'detached'.

*2.3 Lack of feeling understood*

*"…I felt very misunderstood, because I think people, although I admit to myself that I was sort of acting out being self-destructive, acting selfishly, it wasn't 'cause I really had any choice. I think if you're in that situation, you feel so bad about yourself, that that's what kind of, it isn't about other people, 'cause other people are like, well didn't you think about how we feel, how your mum feels? And I'm like, but if you've never felt that way, you can't see beyond you and your own existence."* (Alice, 24 years)

Above, Alice describes how those around her ask Alice why she did not consider the feelings of those who cared for her when she was experiencing her crisis and 'acting out'. From this, Alice explains that these questions indicated to her that people did not recognise her mindset at the time which contributed to her feelings of being misunderstood by those around her at the time. In these

comments, Alice indicates a belief that she was alone in the feelings she was experiencing, and this may have contributed to her feeling unsupported.

_3 Emotional secrecy_

*'… I remember my parents would call me and be like, 'oh how are you doing, are you okay?' And I'd sit there in tears and be, like, yes I'm absolutely fine, Uni is going really well, everything is great.' (Margaret, 25 years)*

Margaret acknowledges the emotional distress she was feeling at the time of her attempt and also that despite being in acute distress, she refused to make her parents aware of her emotional state. Instead, it was more important to Margaret for her parents to believe she was happy, and so she overcompensated for her distress to ensure her parents believed she was thriving ('everything is great'). The non-disclosure of her distress prevented Margaret from getting the emotional and potentially instrumental support she needed at the time to maintain her safety. Through chronic stress, individuals who do not use coping tools such as venting to friends can succumb to their distress leading to poor mental health or suicide.

3.1 Anticipated stigma

Below, Chloe describes her anticipation of negative stigma should she disclose her mental health difficulties to her friends.

*'I did feel that I couldn't really, really talk with anyone about it because I was scared to let them see this part of me and for them to know that this is how I actually am. I felt like I was a fraud and if they found out, they would leave me so I didn't want to be like abandoned, I guess, by them.'* (Chloe, 23 years)

Chloe's anticipation of her friends' reactions to her mental health difficulties invoked fear that she may become a pariah. This suggests Chloe believed that losing her social network would be more

painful than the distress she was feeling already, which illustrates that mental health stigma prevented Chloe from gaining help from those around her.

3.2. Autonomy

*"…I was really concerned that if I went [to hospital], they'd try and section me or I'd lose my autonomy over my own decisions and I'd have this, like, freedom of being able to be at Uni taken away from me. So, I just, I just kind of stayed in my room with my friend and we, like, plastered up my arm* [laughter]."

(Margaret, 25 years)

Margaret describes concern about being admitted to hospital if she sought professional help. Given the self-described extent of Margaret's injuries, this concern for maintaining her autonomy over-ruled seeking help for her physical safety. Instead, Margaret is focused on life remaining unchanged following her suicide attempt, where she still has the freedom to make choices and attend university. This suggests that Margaret potentially believed that attending hospital would have a significant, negative, long-term impact on her life.

*<u>4 Personality traits</u>*

4.1 Socially Prescribed Perfectionism

*"…I've always felt like I need to perform really well and that my value is derived from my work or from physical that I can do good things I still would feel that like they view me a certain way because I do certain things and if I don't manage to do that then, I guess other times my perception was that they will stop bothering me or they will give up on me or they will be disappointed and I don't want to disappoint them."* (Chloe, 23)

The above quote describes the thinking patterns commonly held by individuals with socially prescribed perfectionism; the need to meet the impossibly high real or perceived expectations one believes someone has of them. From this quote, Chloe is discussing her family. She describes her belief that her worth to others is contingent on her abilities; that there is an expectation from others

that she must 'do good things'. Chloe also shows some concern about losing these relationships ('they will stop bothering with me or they will give up on me'). This not only suggests that Chloe feels she has disappointed or failed her family in the past but also that they have not forgiven her and that their love and care for her is limited. As such, Chloe demonstrates here that her capability to do well in her work is imperative in order to keep those she cares about in her life or else be abandoned by them.

*4.2 Anxiety*

*"…By the August point I knew I had to have that year out, and I was a little bit worried, 'cause I'd never been in the situation before where a doctor's just said to you, you're not going back to uni, like, you can't, you can't live your life, you're not fit to do it."* (Alice, 24 years)

Alice describes the fear of the unknown here and the insecurity instilled by her doctor. The various statements Alice recalls the doctor saying when informing her she would not be returning to university that year may reflect the level of distress she felt in the situation and feelings of uncertainty about what she was 'fit to do'.

5. Social transition

*"…Any time she showed some emotional distress, I would* [visit her]*, I'd spend the night, and this was in the initial days of my first year at university, at the time when you do make connections. So I was building a slight rapport with my flatmates at the time, but I was mainly removing myself from the situation by calling to any request she had."* (Aidan, 20 years)

Aidan describes how instead of helping himself assimilate into his new environment at university, he focused on supporting his partner instead. He shares his awareness that this period of time was critical in developing new relationships and indicates some frustration in his actions. Here, Aidan demonstrates that when in a social transition, surrounded by others in a similar circumstance (i.e.,

moving away to university for the first time), it is unhelpful for one to ‘remove [themselves] from the situation’ if they want to form new relationships and ease the transition.
